# Trigeminal Pain and Its Distribution in Different Trigeminal Nerve Branches

**DOI:** 10.5812/aapm.3764

**Published:** 2012-04-01

**Authors:** Maria Nikolaos Piagkou, Panagiotis Skandalakis, Giannoulis Piagkos, Theano Demesticha

**Affiliations:** 1 Department of Anatomy, Medical School, University of Athens, Athens, Greece

**Keywords:** Trigeminal Neuralgia, Pain

Dear Editor,

We read with great interest the recent article by Bangash et al. regarding pain distribution in the different trigeminal nerve (TN) branches in patients with trigeminal neuralgia (TGN) (1). Pain of the 5th cranial nerve is a sudden, unilateral, brief, stabbing, recurrent neuropathic pain in the distribution of one or more of the TN branches. The peak age of onset is between the 5th and 8th decades of life (1-3) and it is thought to be related to neurovascular compression (4).

The authors maintain that this condition is uncommon in people younger than 30 years of age and the most common cause of compression is thought to be venous nerve compression, either alone or in association with arterial nerve compression (4). This concurrence supports the cardinal rule of subjecting patients under 40 years of age who complain of neuralgia-like pain in their face, to a detailed neurological assessment, to exclude associated diseases such as multiple sclerosis (3, 5, 6). Reviewing the literature, some reports demonstrated a male predominance (1, 5, 6) while others showed a predominance of females at various ratios (2, 7, 8). The right side of the face is the most commonly effected due to the narrower foramina rotundum and ovale (7, 9).

The mandibular division is the most commonly involved (inferior alveolar nerve, buccal and mental nerves), followed by the maxillary (infraorbital nerve) and ophthalmic divisions. Bilateral involvement has not been reported. Some authors have described an atypical TN involving pain in both the second and third divisions of the nerve (1, 10). Nevertheless, research should be aimed at understanding the exact reasons for the involvement of the effected side and branches.
